# Randomized Controlled Trial to Assess the Feasibility of a Novel Clinical Decision Support System Based on the Automatic Generation of Alerts through Remote Patient Monitoring

**DOI:** 10.3390/jcm13195974

**Published:** 2024-10-08

**Authors:** Irene Alcoceba-Herrero, María Begoña Coco-Martín, José María Jiménez-Pérez, Luis Leal-Vega, Adrián Martín-Gutiérrez, Carlos Dueñas-Gutiérrez, José Pablo Miramontes-González, Luis Corral-Gudino, Flor de Castro-Rodríguez, Pablo Royuela-Ruiz, Juan Francisco Arenillas-Lara

**Affiliations:** 1 Applied Clinical Neurosciences Research Group, Department of Medicine, Dermatology and Toxicology, University of Valladolid, 47005 Valladolid, Spain; irene.alcoceba@uva.es (I.A.-H.); luis.leal@uva.es (L.L.-V.); adrian.martin.gutierrez@uva.es (A.M.-G.); jfarenillas@saludcastillayleon.es (J.F.A.-L.); 2 Department of Nursing, University of Valladolid, 47005 Valladolid, Spain; jose.maria.jimenez@uva.es; 3 Department of Internal Medicine, University Clinical Hospital of Valladolid, 47003 Valladolid, Spain; jduenas@saludcastillayleon.es; 4 Department of Internal Medicine, Rio Hortega University Hospital, 47012 Valladolid, Spain; jpmiramontes@saludcastillayleon.es (J.P.M.-G.); lcorral@saludcastillayleon.es (L.C.-G.); 5 Emergency Medical Services Direction, SACyL, 47006 Valladolid, Spain; fcastro@saludcastillayleon.es; 6 Technical Direction of Primary Care, SACyL, 47006 Valladolid, Spain; proyuelar@saludcastillayleon.es; 7 Department of Neurology, University Clinical Hospital of Valladolid, 47003 Valladolid, Spain

**Keywords:** telemedicine, decision support systems, clinical, monitoring, physiologic, wearable electronic devices, COVID-19

## Abstract

**Background/Objectives**: Early identification of complications in chronic and infectious diseases can reduce clinical deterioration, lead to early therapeutic interventions and lower morbidity and mortality rates. Here, we aimed to assess the feasibility of a novel clinical decision support system (CDSS) based on the automatic generation of alerts through remote patient monitoring and to identify the patient profile associated with the likelihood of severe medical alerts. **Methods**: A prospective, multicenter, open-label, randomized controlled trial was conducted. Patients with COVID-19 in home isolation were randomly assigned in a 1:1 ratio to receive either conventional primary care telephone follow-up plus access to a mobile app for self-reporting of symptoms (control group) or conventional primary care telephone follow-up plus access to the mobile app for self-reporting of symptoms and wearable devices for real-time telemonitoring of vital signs (case group). **Results**: A total of 342 patients were randomized, of whom 247 were included in the per-protocol analysis (103 cases and 144 controls). The case group received a more exhaustive follow-up, with a higher number of alerts (61,827 vs. 1825; *p* < 0.05) but without overloading healthcare professionals thanks to automatic alert management through artificial intelligence. Baseline factors independently associated with the likelihood of a severe alert were having asthma (OR: 1.74, 95% CI: 1.22–2.48, *p* = 0.002) and taking corticosteroids (OR: 2.28, 95% CI: 1.24–4.2, *p* = 0.008). **Conclusions**: The CDSS could be successfully implemented and enabled real-time telemonitoring of patients’ clinical status, providing valuable information to physicians and public health agencies.

## 1. Introduction

The emergence of new pandemics is a growing global health concern. Based on the record of major epidemics over the last four centuries due to plague, smallpox, cholera, typhoid or variants of respiratory viruses, the estimated probability of a future pandemic with an impact similar to COVID-19 is 2% every year and the risk of intense outbreaks of pandemics could increase threefold in the coming decades [1].

According to the World Health Organization (WHO), these new pandemics could be caused by an influenza virus, a new coronavirus or a new pathogen that we do not even know about yet, which the WHO calls Disease X [2]. In this sense, given that pandemic outbreaks are increasingly possible in the near future, the prevention and control of diseases with pandemic potential must be prioritized, both locally and globally. To this end, WHO Member States are working towards an international agreement on pandemic preparedness and response, with the aim of strengthening capacities for future pandemics, improving warning systems, data sharing, research and the production and distribution of health equipment, and promoting new procedures to control pandemic events [3].

The difficulties of in-home monitoring of patients with infectious diseases have promoted the development and implementation of digital health systems based on Artificial Intelligence (AI) and machine learning to improve patient monitoring without overloading healthcare professionals [4,5]. In the process of healthcare digitalization, real-time monitoring of patient’s health has been established as a basic pillar, through patient self-reporting of symptoms or mood and monitoring of vital signs, such as heart rate (HR), body temperature (BT), blood pressure (BP), and respiratory rate (RR), with wearable devices such as smartwatches [2,6,7].

The creation of clinical decision support systems that integrate different telemedicine-based monitoring systems has the potential to improve clinical outcomes, increase continuous follow-up, strengthen social separation, detect complications, optimize resource use, and reduce complications [8,9,10]. As a response especially in primary care, a digital transformation of healthcare is being driven, using CDSS systems for better health management in case of a future infectious-contagious disease, facilitating care in rural areas, increasing follow-up in patients at high risk of complications, and empowering patients and their relatives in the active management of their health [11,12,13]. Therefore, healthcare platforms need to be designed, implemented, and evaluated, as they face continuous challenges, such as the variability of the wireless channel determined by changes in body posture, the wide variety of non-validated sensors for clinical monitoring, and the large amount of data produced that cannot overload professionals [10,13,14].

Therefore, we set out to design and evaluate a novel CDSS based on the automatic generation of alerts through remote patient monitoring. Our primary objective was to assess the feasibility of the system, while our secondary objective was to determine the patient profile associated with the likelihood of severe medical alerts.

## 2. Materials and Methods

### 2.1. Study Design

This study is a prospective, multicenter, open-label randomized clinical trial. Patients diagnosed with COVID-19 in mandatory home isolation from November 2021 to January 2022 were screened. Patients who agreed to participate in the study were assigned to one of the two study arms by simple randomization using computer-generated random numbers. The interventions assigned to each group are described in Table 1. The control group received conventional telephone follow-up by primary care health professionals, and the patient could self-report their clinical symptoms on the mobile app three times a day (every 8 h via push notification). The case group received conventional telephone follow-up by primary care health professionals, could self-report their clinical symptoms via the mobile app three times a day, and also had their vital signs monitored remotely and 24 h a day using a pulse oximeter and a commercial wearable device whose measurement reliability was ensured in an earlier phase of the study in which 4490 measurements from 68 COVID-19 inpatients were compared with those from a vital signs monitor used as a gold standard (CARESCAPE™ B450, GE Healthcare, Chicago, IL, USA).

Moreover, the monitored vital signs from patients in the case group were continuously and remotely visualized on a dashboard so that primary care professionals were aware of the patient’s evolution. The system produced alerts when changes were detected that exceeded previously defined severity thresholds in both vital signs and clinical symptomatology. Alerts were classified as technological when they were automatically managed and resolved by the system through IA algorithms, without notifying healthcare professionals. These included alerts resulting from incorrect monitoring or that were deactivated by the patient through verification questions received in the application. These questions addressed issues that could invalidate proper monitoring, such as whether the patient was exercising at the time the alert was generated. Medical alerts classified as mild or moderate were notified via a message on the web platform for health center professionals, while serious alerts were handled immediately via a phone call to the Emergency Health Service (EHS). This randomized controlled trial was registered on clinicaltrials.gov (NCT04802018), follows the CONSORT 2010 statement (Supplementary Table S1), and was conducted according to the previously published protocol [15].

### 2.2. Study Setting

During 6 weeks from November to December 2021, we consecutively sampled all patients from primary health care centers in the East and West health areas of Valladolid (Castile and Leon, Spain). Patients were recruited and followed up by primary healthcare professionals from the two health areas of Valladolid: from 8 urban primary health care centers in the Western area and from 17 in the Eastern area (8 urban and 9 rural). To achieve a random allocation of patients, each patient was assigned a three-digit numerical code indicating the consecutive order of inclusion and was randomly included in the case or control group through an automatic selection table.

Inclusion criteria for study participation involved a positive diagnosis of COVID-19 by polymerase chain reaction (PCR) or antigen testing, a time window of ≤6 days between positive COVID-19 diagnosis and inclusion, internet access at home, possession of a Smartphone with Android^®^ operating system (Google LLC, Mountain View, CA, USA), willingness to electronically sign the informed consent form required to participate in the study, and availability to wear the monitoring devices if randomly assigned to the case group. In contrast, exclusion criteria were age below 16 years, lack of digital skills to use the “Home app” mobile application, hospitalization or significant clinical progression that could lead to hospital admission between the date of COVID-19 diagnosis and the date of inclusion in the study, and disabling upper limb pathology or cognitive impairment that may preclude participation in the study.

**Figure 1:**
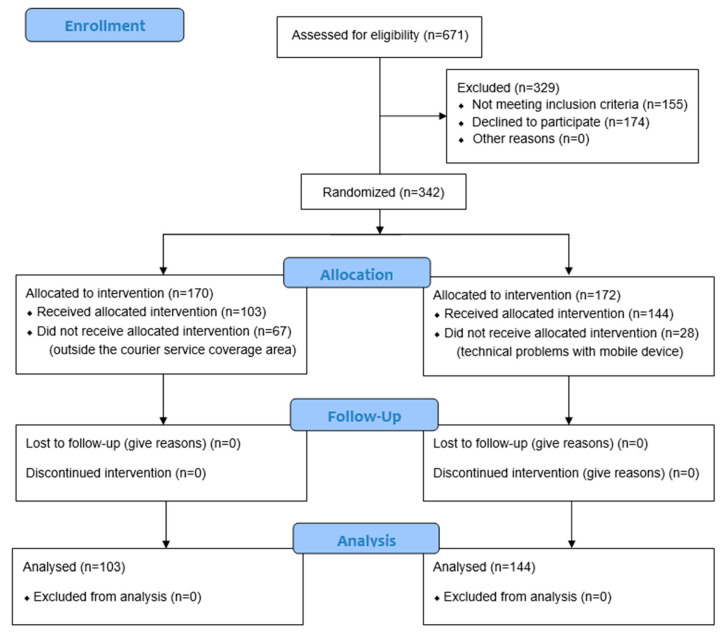
CONSORT 2010 flow diagram.

As represented in the flow chart in Figure 1, of the total number of patients screened to participate in the study (*n* = 671), 51% (*n* = 342) belonged to the intention-to-treat group, patients who met the selection criteria and were randomized to participate in the study (170 cases and 172 controls). In the end, 247 individuals, 103 cases, and 144 controls completed the entire protocol of the study.

### 2.3. Study Variables

The following variables were collected from each patient:

- Baseline variables under study were age, sex, date of positive COVID-19 diagnosis, diagnostic method (polymerase chain reaction or antigen test), COVID-19 vaccination schedule (number of vaccinations and type), and comorbidities with a high risk of severe disease progression.
- Variables of the conventional clinical assessment questionnaire carried out by telephone by primary care professionals for home follow-up of patients with COVID-19.
- Clinical symptoms self-reported by patients using the mobile phone application for subsequent visualization by healthcare professionals: cough, fever, chest pain, dyspnea, vomiting, and diarrhea. Alterations in clinical symptoms produced alerts and the two groups were compared in terms of the number of alerts received and the ability of the system to resolve them autonomously.
- Vital signs were monitored in real-time in the case group using the Bakeey E66 smartwatch (Shenzhen Yisi Technology Co. Ltd., Guangdong, China), which measures HR in beats per minute (bpm), RR in breaths per minute (bpm), and Tª in degrees Celsius (°C). In addition, the Wellue FS20F pulse oximeter (Wellue, Los Angeles, CA, USA) monitored blood oxygen saturation (SpO_2_) in percent (%). The system produced mild-moderate alerts for HR greater than 100 bpm, RR greater than 22 bpm, BT greater than 38 °C, or SpO_2_ less than 94%. It also produced severe alerts for HR greater than 120 or less than 50 bpm, RR greater than 30 or less than 9 bpm, BT greater than 39 °C, or SpO_2_ less than 92%.
- Prognostic variables: were assessed 30 days after the date of positive COVID-19 diagnosis. To reduce the risk of error, the evaluation was carried out by an external clinical specialized committee on infectious diseases. Primary variables were progression to pneumonia, need for intensive care unit (ICU) admission, need for invasive mechanical ventilation, and mortality rate. Secondary outcomes were hospital admission rate, mean hospital stay, the occurrence of major vascular events, and the economic cost of hospital care.

### 2.4. CDSS

**Figure 2:**
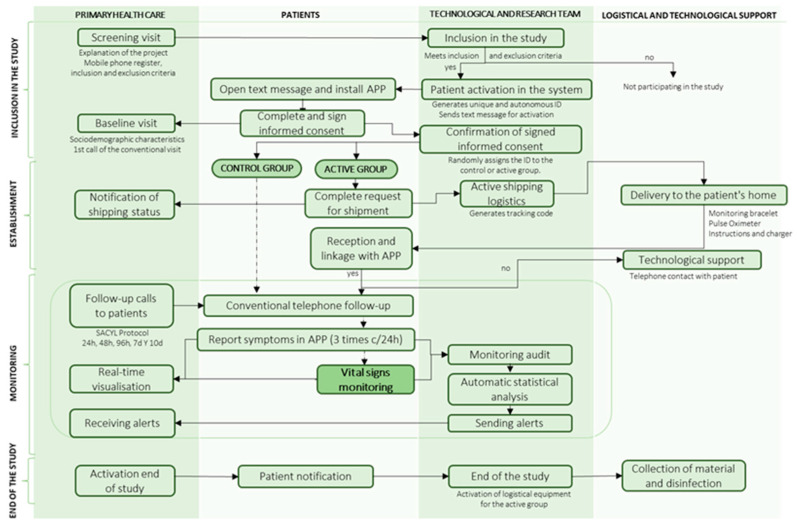
Flow diagram for study management.

The conduct of the clinical trial is divided into 4 phases, as shown in Figure 2:

In the inclusion phase, a screening visit was carried out in which nurses and primary care doctors explained the project and recorded the patient’s telephone number and inclusion and exclusion criteria on the dashboard. If they met the screening criteria, they were automatically activated in the system and received an SMS to download the digital application “Home”, where they signed the informed consent form. The healthcare professional recorded their socio-demographic characteristics and made the first baseline phone call of the conventional primary care protocol. The system checked that the documentation was signed and assigned the patient a random identifier to belong to the case or control groups.

In the set-up phase, patients in the case group filled in their logistical details to receive the monitoring devices at home. They received the pulse oximeter and the smartwatch to monitor vital signs and then had to link them to the “Home” health app. Both patients and healthcare professionals were provided with technological, logistical, or clinical support for questions via the Chat-box or by telephone assistance for 12 h a day.

In the follow-up phase, patients in both groups reported their clinical symptoms three times a day using the app and received conventional phone calls according to the primary care protocol. In addition, in the case group, vital signs were monitored remotely 24 h a day during home quarantine using a wearable device. SpO_2_ was measured 3 times a day when the app sent a notification for the patient to put on the pulse oximeter. Healthcare professionals could view the clinical evolution of vital signs and symptoms via the dashboard and receive moderate to mild and severe alerts. The system filters out false alerts by patient confirmation, and confirmed severe alerts automatically triggered the EHS.

In the final phase, healthcare professionals activated the end of the study and the patient was automatically notified. For patients in the case group, the logistic service for device collection and disinfection was activated.

### 2.5. Data and Information Management in the Institutions

**Figure 3:**
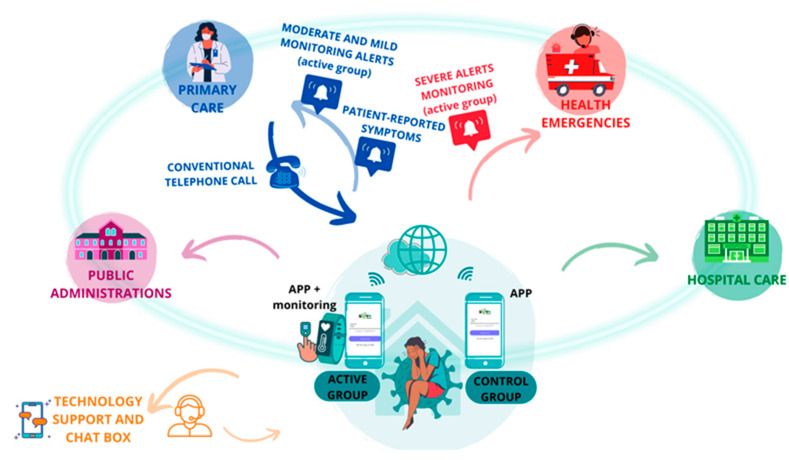
Interrelationship between patients and institutions.

The patient was at the center of the system, reporting symptoms three times a day on an app, and those in the case group had their vital signs monitored remotely (Figure 3). All the data generated were processed by AI algorithms, which triggered the generation of medical alerts produced by altering vital signs, clinical alerts derived from the symptomatology reported by the patient, or technological alerts produced by the incorrect functioning of the technological devices.

### 2.6. Statistical Analysis

The statistical package Stata 15 (StataCorp College Station, TX, USA) was used to analyze the data, and sociodemographic characteristics and comorbidities were described through descriptive analysis. The analysis of variance (ANOVA) technique was applied to determine the existence of significant differences between the two groups, the Kaplan–Meier estimator and the Cox proportional hazards model to identify possible predictive factors. Likewise, the association of the variables was performed using Pearson’s Chi-square test, Fisher’s exact test, and the degree of association using Cramer’s test. Sociodemographic characteristics, baseline variables, and previous pathologies were correlated with the severity of the alerts. In addition, logistic regression using Odd Ratio (OR) with 95% confidence intervals (95% CI) was performed to determine the probability of having a severe alert according to a given characteristic. A statistical significance level of *p* < 0.05 was assumed.

### 2.7. Ethical Considerations

The study was approved by the Medical Research Ethics Committee of the Valladolid East Health Area on 18 February 2021 (CASVE-NM-21-508) and adhered to the principles of the Declaration of Helsinki. The information obtained from the health applications and monitoring devices was guarded by the research team while respecting the anonymity of the participants by assigning identification codes. The system guaranteed all cybersecurity requirements, the study was approved by the Office of the Public Information Security Service of the General Directorate of the Community of Castilla y León on 28 December 2021 and was registered in Clinical Trials on 16 March 2021 (NCT04802018).

## 3. Results

### 3.1. Description of the Study Sample

A total of 342 patients were included in the study, of whom 170 were randomly assigned to the case group and 172 to the control group. Of these, 247 persons completed the full protocol, of whom 103 belonged to the intervention group (41.7%) and 144 to the control group (58%). Sample loss in the case group was mainly due to problems with the delivery of monitoring devices to patients’ homes due to the limited coverage offered by the courier service delivering them under the COVID-19 contingency law.

Table 2 presents the baseline characteristics of the study sample. It should be noted that the analysis of association of baseline characteristics between the study groups only revealed statistically significant differences in terms of gender (*p* = 0.04). When analyzing comorbidities, the most prevalent were dyslipidemia, obesity, and hypertension.

### 3.2. Clinical Prognostic Variables and the Evolution of Both Groups

Primary and secondary prognostic variables were assessed in the 247 patients who completed the full study protocol. None of the patients in both groups experienced either a primary or a secondary clinical endpoint.

### 3.3. Emission and Management of Alerts

**Figure 4:**
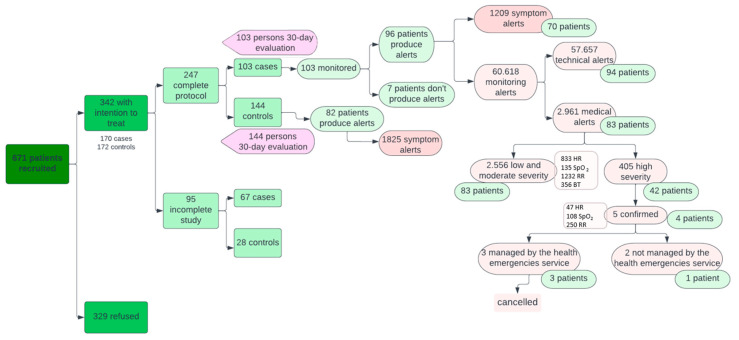
Interrelationship between patients and institutions.

As for alerts (Figure 4), 82 patients in the control group issued more symptomatology alerts than the case group (1825 versus 1209; *p* < 0.05). These alerts from the Home app were displayed by primary care professionals on the system’s dashboard during their working hours and therefore did not represent a significant work overload for them.

On the other hand, of the 60,618 alerts coming from wearable devices (in the active group only), 57,657 (94.86%) were technological alerts resolved autonomously by the CDSS and 2961 (5.14%) were medical alerts managed by healthcare staff. Of the medical alerts, 2556 were mild or moderate (883 for HR, 135 for SpO_2_, 1232 for RR, and 356 for BT) and 405 severe (47 for HR, 108 for SpO_2_, and 250 for RR). Mild and moderate alerts from the devices were treated in the same way by primary care professionals as symptom alerts from the Home app, while severe alerts were automatically escalated to EHS once they were confirmed by the patient. This happened in five cases where the EMS contacted the patient via a phone call, checked the patient’s situation, and deemed that no further care was needed. The remaining 400 severe alerts (not confirmed by the patient) were managed by the primary care professionals by checking on the system’s dashboard that they were transitory alterations that did not require additional support and verifying the patient’s clinical stability in 67% of the cases via a telephone call. In this way, there were no false positives in terms of serious alerts resulting in unnecessary escalation to the EHS.

### 3.4. Baseline Factors Associated with Medical Alert Production

Concerning medical alerts, men generated more total alerts (*n* = 1579) than women (*n* = 1382). Women produced more RR and HR alerts (*p* < 0.05), while men produced more SpO_2_ and BT alerts (*p* < 0.05). Regarding age, there were more HR alerts in those younger than 30 years and 30–45 years (*p* < 0.05). Likewise, more RR alerts were found in the group aged 30–45 years and SpO_2_ in the two older age groups, in those aged 46–65 and those over 65 years (*p* < 0.001). On the other hand, when correlating comorbidities with the presence of alerts, hypertension and dyslipidemia were related to SpO_2_ and BT alerts (*p* < 0.05); obesity with SpO_2_ and RR alerts; corticoids and asthma with RR (*p* < 0.05).

The Table 3 shows the bivariate analysis of baseline variables potentially associated with medical severe alert generation in both study groups. Severe alerts were sensor-dependent, with a higher number of alerts in SpO_2_ and RR (*p* < 0.05). In addition, more severe alerts occurred in patients over 65 years of age (*p* < 0.05) and in patients diagnosed with asthma or treated with corticosteroids (*p* < 0.05).

When variables that were significantly associated with the likelihood of triggering a severe alarm in the bivariate analysis were included in a logistic regression model, controlled for gender, the following variables emerged as independently associated with an increased likelihood of triggering a severe alarm: having asthma (OR: 1.74, 95% CI: 1.22–2.48, *p* = 0.002) and taking corticosteroids (OR: 2.28, 95% CI: 1.24–4.2, *p* = 0.008).

## 4. Discussion

Our novel CDSS demonstrated the ability to continuously and remotely monitor all vital signs of the patient in home isolation and automatically alert primary care and emergency staff in case of severe alteration. Given the benign course of the disease in our sample, probably due to a high vaccination rate at the time of the study, we were unable to observe whether this increased protection could translate into significant changes in prognostic variables and healthcare cost savings [16]. After analyzing the quantity and quality of real-time information provided by the CDSS, we can speculate that, if this system had been available during the first pandemic wave, there might have been differences in prognostic variables between the two groups.

While the present study monitored vital signs and symptomatology, other clinically relevant studies only assessed some variables, as shown in the systematic reviews by Kondylakis et al. [17] and Takahashi et al. [18], mainly SpO_2_ and RR. Similarly, the study by Xu et al. [19] or Faris et al. [20] implemented telemedicine systems focused on self-reporting of symptoms by the patient and automatic visualization of symptomatology by healthcare professionals, improving the monitoring and management of personal resources. The novelty of the present CDSS is that it implements all forms of telemedicine, self-reporting of symptoms in an application, telemonitoring of vital signs in real time, and conventions calls by primary care professionals. Despite increasing the amount of information and individualized monitoring, it did not increase the workload of the professionals because the system used AI to manage the data and produce alerts automatically. In relation to the use of these technologies, studies that focus on follow-up at hospital discharge stand out, but it is essential to apply CDSS in the early detection of patient deterioration at home, as it can reduce admissions, minimize complications, improve patient satisfaction and health outcomes, and minimize healthcare costs [17,18,21].

Furthermore, most of the articles in the systematic review by Vindrola et al. [22] were led by secondary care professionals; however, it has been shown that joint management of systems by primary and secondary care allows the implementation of more effective systems for remote monitoring at home, as has happened in our study. Some clinical characteristics and comorbidities were significantly associated with a higher prevalence of alerts in this study, coinciding with those reported as risk factors for hospital admission or COVID-19 mortality in other studies [23,24]. Wurzer et al. monitored the same vital signs at home, but only in patients with risk factors, without producing alerts, and it was useful to detect worsening [25]. In addition, patients in rural areas are at higher risk of a more severe course due to COVID-19 [26] and the study was conducted in both urban and rural areas.

The literature shows that there is a clear need for studies using telemedicine, to plan for future pandemics and to monitor infection diseases, which could benefit from this CDSS as it would allow better patient monitoring, early detection of complications by issuing alerts, improved multidisciplinary care, reduced professional overload, a more case attitude of patients, and better resource management [26,27]. The study of these systems will allow us to be better prepared for future pandemics, promoting health equity, communication, community participation, collaborative networks, and surveillance [28].

The present study has some identified limitations: at the time of implementation of the study, most of the sample was vaccinated (80% of the vaccinated population), which conditioned the assessment of prognostic variables since the patients did not progress to severe stages requiring hospitalization; therefore, the primary and secondary variables were not altered in any patient. The delay in launching the study was due to difficulties in complying with data protection and digital security policy requirements. Moreover, the loss of the initial sample of patients who did not complete the protocol may generate a bias in the analysis of the data that affects the representativeness of the sample. Despite the above, the designed CDSS has shown to be useful for the exhaustive monitoring of other pathologies or pandemics.

## 5. Conclusions

The CDSS provided real-time information, accessible online to the team of healthcare professionals, and was able to automatically produce and manage more information and more alerts than the conventional system; the caseload was better controlled by monitoring without overloading the professional. The system allowed for individualized care and provided continuous, multidisciplinary monitoring through the constant linkage between primary care, emergency health care, and inpatient professionals. The system can be applied to future pandemics or infectious diseases.

Regarding alerts, men produced more SpO_2_ and BT alerts, more SpO_2_ alerts were recorded in the two older age groups, and obese people issued more SpO_2_ and RR alerts. The characteristics of the patients that produced the most severe alerts were age over 65 years, diagnosis of asthma, and treatment with corticosteroids.

## Figures and Tables

**Table 1:** Treatment of intervention and control group.

Control Group	Intervention Group
− Conventional primary care telephone follow-up (24 h, 48 h, 96 h, 7 d, 10 d)	− Conventional primary care telephone follow-up (24 h, 48 h, 96 h, 7 d, 10 d)
− Patient self-reporting of clinical symptoms every 8 h via the Home mobile app	− Patient self-reporting of clinical symptoms every 8 h via the Home mobile app
	− Real time telemonitoring of patients’ vital signs (HR, RR, BP, BT, SpO_2_)

BP: Blood pressure; BT: Body temperature; HR: Heart rate; RR: Respiratory rate; SpO_2_: Blood oxygen saturation.

**Table 2:** Baseline characteristics of patients.

Variables	Cases *n* (%)	Controls *n* (%)	*p*-Value
Men Women	52 (50.5%) 51 (49.5%)	54 (37.5%) 90 (62.5%)	0.04 *
<30 years old 30–45 years 46–65 years >65 years	13 (12.6%) 39 (37.9%) 46 (44.7%) 5 (4.9%)	28 (19.4%) 55 (38.2%) 57 (39.6%) 4 (2.8%)	0.43
COVID-19 PCR test positive COVID-19 antigen test positive	69 (67.0%) 34 (33.0%)	96 (66.7%) 48 (33.3%)	0.96
COVID-19 vaccinated	69 (67.0%)	102 (70.8%)	0.51
High blood pressure Diabetes mellitus Obesity Dyslipidemia Atrial fibrillation Myocardial infarction Asthma Bronchitis COPD Stroke Liver disease Neoplasia Chronic renal failure	10 (9.7%) 3 (2.9%) 10 (9.7%) 17 (16.5%) 1 (1.0%) 0 (0.0%) 8 (7.8%) 0 (0.0%) 2 (1.9%) 0 (0.0%) 1 (1.0%) 0 (0.0%) 0 (0.0%)	11 (7.6%) 3 (2.1%) 13 (9.0%) 15 (10.4%) 4 (2.8%) 1 (0.7%) 10 (6.9%) 1 (0.7%) 0 (0.0%) 3 (2.1%) 0 (0.0%) 4 (2.8%) 1 (0.7%)	0.57 0.68 0.86 0.16 0.32 0.4 0.81 0.4 0.09 0.14 0.24 0.09 0.4
Corticosteroids Immunosuppressive biological therapy	3 (2.9%) 0 (0.0%)	5 (3.5%) 5 (3.5%)	0.8 0.06

COPD: Chronic Obstructive Pulmonary Disease; COVID-19: Coronavirus disease 2019; PCR: Polymerase chain reaction; *: statistical significance (*p* < 0.05).

**Table 3:** Correlation of severe alerts with baseline characteristics of patients.

Variables	*n*	Chi-Square *p*-Value	Cramér’s V
Men Women	225 180	0.36	0.02
<30 years old 30–45 years 46–65 years >65 years	9 78 304 14	<0.001	0.08
COVID-19 PCR test positive COVID-19 antigen test positive	356 49	0.72	0.01
HR SpO_2_ RR	47 108 250	<0.001	0.33
High blood pressure Diabetes mellitus Obesity Dyslipidemia Atrial fibrillation Asthma COPD	27 4 22 113 3 56 3	<0.001 0.11 0.21 <0.001 0.47 <0.001 0.90	0.15 0.03 0.03 0.07 0.01 0.09 0.01
Corticosteroids	20	<0.001	0.33

COPD: Chronic Pulmonary Obstructive Disease; COVID-19: Coronavirus disease 2019; HR: Heart rate; PCR: Polymerase chain reaction; RR: Respiratory rate; SpO_2_: Blood oxygen saturation.

## Data Availability

Dataset available on request from the author.

## Supplementary Materials

The following supporting information can be downloaded at: https://www.mdpi.com/article/10.3390/jcm13195974/s1, Table S1: CONSORT 2010 checklist of information to include when reporting a randomised trial. Reference [29] is cited in the Supplementary Materials.
